# Does institutional quality moderate the relationship between corporate governance and stock liquidity? Evidence from the emerging market of Pakistan

**DOI:** 10.3389/fpsyg.2022.912796

**Published:** 2022-09-08

**Authors:** Shuaib Ali, Wu Zhongxin, Zahid Ali, Guo Fei, Muhammad Abir Shahid Chowdhury

**Affiliations:** ^1^School of Management, Hainan University, Haikou, China; ^2^Department of Management Sciences and Commerce, University of Malakand, Chakdara, Pakistan; ^3^School of Accounting, Zhongnan University of Economics and Law, Wuhan, China; ^4^School of Economics and Management, China University of Geosciences, Wuhan, China

**Keywords:** Institutional Quality, corporate governance, stock liquidity, PCA, Pakistan

## Abstract

The main aim of this study was to empirically analyze whether Institutional Quality moderates the relationship between corporate governance and stock liquidity through the light of agency and information asymmetry theory. To the best of our knowledge, this is the first finance study. The sample consists of 230 non-financial firms listed on the Pakistan stock exchange during the period of 2009–2019. We used an instrumental variable approach and our new Institutional Quality index composed of world governance indicators and a corporate governance index, developed *via* principal component analysis, to demonstrate a relationship between corporate governance and stock liquidity and check the moderating role of Institutional Quality by following the resources complementary phenomenon. Our results show a significant, positive relationship between the corporate governance index and stock liquidity, suggesting that well-governed firms have high liquidity. The results show that the Institutional Quality index has a positive moderating impact on the relationship between corporate governance and stock liquidity, suggesting that corporate governance in Pakistan is weak. Our results are robust to a series of endogeneity checks using alternative proxies of stock liquidity.

## Introduction

The financial assets’ liquidity has been recognized as an essential component of the smooth operation of the capital markets. It supports market contestants to meet sudden financial requirements without any unbearable losses. Liquidity plays an essential role in asset pricing and has recognized the great interest of researchers worldwide. A completely liquid market may immediately turn every quantity of a particular stock in that market into cash and at no expense. To find ways to improve the liquidity of the shares, it can be either regulators or financial analysts to build a significant focus on academic and professional concerns.

Regulators contribute to protecting minority investors, apart from the possibility of expropriation, and facilitating their robust business participation that raises liquidity marginally (Brockman and Chung, 2008a,b). Biswas (2020) claimed that an increase in corporate governance quality will enhance stock liquidity. Shareholders expect to gain because they face volatility and transactional costs by selling their shares in the market (Amihud and Mendelson, 2006).

The company governance notion has been established in numerous ways. Tricker and Tricker (2015) categorized concepts according to five different perspectives, namely, organizational, behavioral, relationship, financial, and social. Most of the studies on corporate governance have been conducted from the institutional perspective, in which owners, the board of directors, and the administration are focused. Corporate governance concepts are based on institutional control and emphasis on governance systems, procedures, and activities (Tricker and Tricker, 2015). Utami et al. (2020) pointed out that ownership structure significantly affects stock liquidity. A perfect example is a description by Sir Adrian Cadbury of corporate governance as the structure that regulates and governs businesses.

Given this claim, there is less empirical evidence of the correlation between interior quality corporate governance and stock liquidity for emerging economies like Pakistan. The quality of corporate governance enhances stock liquidity in the United States (Chung et al., 2010). However, the results are bound for a short period (2001–2004), agreeing with the previous definition of the Sarbanes–Oxley Act of 2002, which might have caused a false association between corporate governance quality and stock liquidity. However, there are no homogeneous emerging countries, with Pakistan being one of them.

North (1991) stated that the formal and informal conduct of people in a country is the Institutional Quality of that region. The standard components are the rules and regulations, the framework for protecting investors and property privileges, and the administrative arrangement of the state. In contrast, the informal component is the natural conduct of the citizens and culture, which has been built in line with the historical pattern of behavior. Hodgson (2006) observed institutions as a social phenomenon because they set the rules for the game, which is obligatory for corporations and organizations to sustain.

This study uses a sample of 230 non-financial firms listed on the Pakistan Stock Exchange (PSX) during the period of 2009–2019. To the best of our knowledge, this is the first finance study to investigate the moderating effect of Institutional Quality on the relationship between corporate governance and stock liquidity. This is also the first study to analyze the relationship between corporate governance and stock liquidity in Pakistan and to establish new Institutional Quality and corporate governance indexes (CGI) using principal component analysis (PCA).

Our study contributes to the literature of Institutional Quality, corporate governance, and stock liquidity in several ways; specifically, this study complements the previous literature of corporate governance and stock liquidity from Malaysia (Foo and Zain, 2010), China (Lei et al., 2013; Wang et al., 2022), Thailand (Prommin et al., 2014), and France (Karmani and Ajina, 2012). However, none of these studies is based on an emerging market like Pakistan.

Firstly Pakistan has highly concentrated firm ownership, with most families holding firms. Most corporate boards are merely “rubber stamps,” with the family owning the bulk of the shares. Pakistani firms rely primarily on bank loans for financing. The public capital market has a passive role in financing as compared to developed markets. As Pakistani firms depend much more on capital market financing than firms in developed countries, stock liquidity plays a different role in Pakistan. Furthermore, its capital market does not efficiently communicate information but instead has weak corporate governance, which results in information asymmetry and agency problems. Therefore, the Pakistan market is significantly less liquid than the United States market and other developed markets.

Second, to the best of our knowledge, this is the first study to analyze the moderating role of Institutional Quality on the relationship between corporate governance and stock liquidity. Our study contributes to the literature by showing how Institutional Quality can moderate this relationship by following the resources complementary phenomenon. Third, our study contributes to the literature by using the PCA for both CGI and Institutional Quality index, which has never been covered before in any study, and the advantages of PCA are explained in the analysis section of the study.

We have used an instrumental variable (IV) approach and established corporate governance and Institutional Quality indexes *via* PCA. We found evidence that Institutional Quality positively moderates the relationship between corporate governance and stock liquidity, suggesting that corporate governance is weak, and by following the resource complementary phenomenon, Institutional Quality positively moderates the relationship between corporate governance and stock liquidity. Our results show a significant and positive relationship between corporate governance and stock liquidity, suggesting that well-governed firms have high liquidity. Our results are robust to a series of endogeneity checks using alternative proxies for stock liquidity.

**FIGURE 1:**
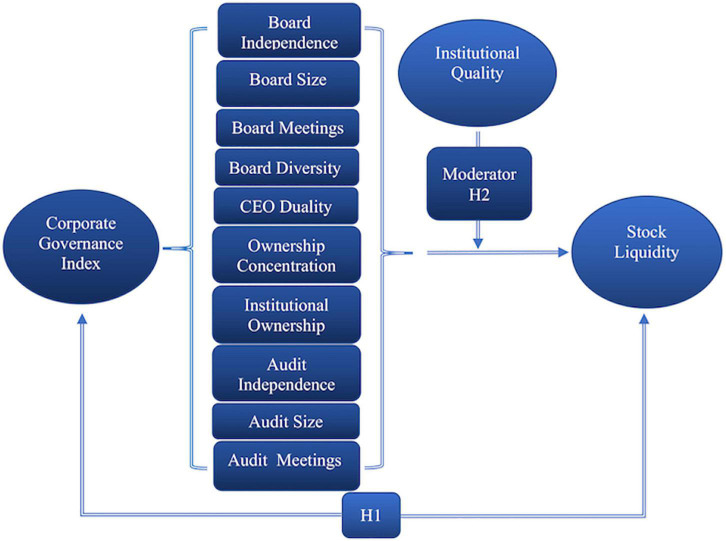
Conceptual framework.

The remaining paper is structured as follows. The Figure 1 shows the conceptual frame work of the study. The “Literature review and hypothesis development” section provides a review of the relevant literature and hypothesis development. The “Materials and methods” section describes the data and research design used to examine corporate governance and stock liquidity. The “Results and discussion” section discusses the results of the study. The “Conclusion” section presents the conclusions, including limitations, future directions, and policy implications.

## Literature review and hypothesis development

### Stock liquidity

Various studies on liquidity have been conducted globally, and various opinions have been documented. The study by Amihud and Mendelson (1986) initially recorded a significant and robust association between stock and illiquidity. Amihud and Mendelson (1986) also indicated the existence of an optimistic and significant relationship between projected income and stock liquidity. The relationship between stock returns and liquidity was analyzed by Nguyen et al. (2021) who pointed out the significant association between expected income and liquidity of equity by using Amihud illiquidity. Liquidity is the ability to trade fast and with rates that are not substantially moving and lead to economic growth (Schwartz et al., 2020).

The relationship between liquidity and information asymmetry was examined for unexpected disclosure occasions of Australian mining firms (Katselas et al., 2021). The author found increased market liquidity with improved transparency in the discussions by reducing the possibilities for the least accomplished stakeholders (Ghazizadeh et al., 2021). As the rise in liquidity in asymmetrical information on the market has been found, insiders can achieve a better result concurrently relative to their trades for liquidity investors. Therefore, it is argued that market liquidity is not inherently diminished by the existence of informed traders (Cornell and Sirri, 1992).

### Corporate governance

Corporate governance value has increased in firms because of the division of management and ownership rights of modern companies. Shareholders’ interests are contradictory to administrators’ interests. The principal-agent concern is due to the diverse interests of the owners in the company’s management and directional issues. Corporate governance has no specific definition; instead, it is seen from various points of view (Smith, 1776).

Rajan and Zingales (1998) explained corporate governance by way of “allocation of ownership, capital structure, managerial incentive schemes, takeovers, board of directors, pressure from institutional investors, product market competition, labor market competition, organizational structure, etc., can all be thought of as institutions that affect the process through which quasi-rents are distributed.” Garvey and Swan (1994) stated that corporate governance is how the organization’s ultimate decision-makers (management) eventually handle those agreements. Shleifer and Vishny (1997) described corporate governance as “how financial providers of companies ensure their investment returns.”

Ghazizadeh et al. (2021) studied the impact of corporate governance on the financial performance of British firms. They found that if the governance mechanisms are chosen so the finances increase. Lien et al. (2005) conducted research on corporate governance and performance at Taiwan family-run firms. They examined the impact of the ownership structure and board features on performance in publicly listed corporations regulated by families. Companies located in East Asia work in a distinctive cultural setting and separate legislative and judicial structures from Western and Europe; these cultural disparities significantly affect the governance success partnerships proposed by the agency and strategic studies.

### Institutional Quality

Many researchers have examined the importance of institutions’ quality, especially the impacts of the legal and regulatory climate on making operational and economic markets. The legal and regulatory framework concerning the protection of property rights, contract compliance, and accounting control standards has been established as importance for better liquidity.

Náplava (2018) stated that Institutional Quality provided accurate circumstances for long-term growth and increased economic performance. It can be seen very prominently in La Porta et al. (1997) argued that the legal code’s roots significantly affect the protection and performance of investors and lenders. They concluded that minor shareholder privileges are linked to poorly established stock markets (mainly in states under French civil law). Agostino et al. (2020) studied the relationship between Institutional Quality and a firm’s productivity. The findings suggest that Institutional Quality enhances the productivity of European firms.

Levine (1998) has observed that countries with legislative and regulatory frameworks prioritizing creditors who collect their business claims have stronger financial intermediaries than those in which the legislation gives much less protection to investors. Çam and Özer (2022) conducted a study on the impact of Institutional Quality on capital structure and investment decisions of the firm. They argued that firms operating in the country having better Institutional Quality enhance their reliance on long-term debt and equity issuance in financing capital expenditure while decreasing short-term debt and equity issuance. Andrianova et al. (2011) highlighted the crucial position of the government as a political entity, which establishes significant commercial monopolies, thus facilitating the rise of global financial markets.

### Corporate governance index and stock liquidity

The scope of internal corporate governance quality in assessing stock liquidity is illustrated in classical studies. Coffee (1991) claimed that major investors endorse internal governance structure because these mechanisms boost stock liquidity, making their exit less expensive. Withstanding this statement, there is insufficient empirical proof for the correlation between internal CG and stock liquidity. For example, the authors argued that board interlocks are positively related to stock liquidity (Mbanyele and Wang, 2022). Chung et al. (2010) illustrated that corporate governance quality increases stock liquidity in American firms.

This throws doubt upon the generalization of the findings to other countries from the United States, in which there are no generally pro-accounting rules and high-frequency liquidity measures. Further studies on corporate governance quality and stock liquidity were conducted in developing countries like Malaysia (Foo and Zain, 2010), China (Lei et al., 2013), Thailand (Prommin et al., 2014), and France (Karmani and Ajina, 2012). Generally, these studies were influenced by small samples and insufficient liquidity measures. For example, Prommin et al. (2014) recorded that strong governance increases stock liquidity over the period in Thailand.

Strengthening corporate governance increases the company’s information transparency and eliminates information asymmetry between insiders (e.g., managers) or external entities (e.g., investors). When less critical asymmetric information, investors are less vulnerable to unfavorable specific risks (Glosten and Milgrom, 1985). Therefore, they offer more liquidity to firms with a robust governance system.

This theoretical argument is supported by several empirical studies showing that firms with better corporate governance have a better information environment and improved liquidity in stock (Glosten and Milgrom, 1985; Prommin et al., 2014; Wahed, 2017). Daadaa (2021) conducted research on corporate governance and stock liquidity, and findings suggest that strong corporate governance will enhance stock liquidity. The author argued that the increase in corporate governance causes a significant increase in the stock liquidity of the firm. The findings suggest 1 SD increase in governance will decrease the illiquidity ratio by 55.97% (Biswas, 2020).

In developing countries like Pakistan, corporate governance tends to be weaker. For all these reasons, findings based on developed countries cannot be extended to emerging markets. Moreover, evidence is mixed and inconclusive from developed countries, with a range of distinctive features. For example, equity markets are much less developed because most companies rely on bank loans. The equity is much less liquid. Therefore, according to the discussion, we hypothesized that

**H1:** Corporate governance index and stock liquidity are positively related.

### Corporate governance index, Institutional Quality index, and stock liquidity

According to the established market analytical studies, corporate governance increases liquidity in the financial markets (Bacidore and Sofianos, 2002; Brockman and Chung, 2003). Ajina et al. (2015) suggested that disclosure level positively affects the French stock liquidity. Weak corporate governance results in an increased asymmetry of information. Liquidity suppliers would impose extra detrimental risk information and thus provide higher information asymmetry features for their efficient bid-ask spread (Chen et al., 2007).

Current finance literature moved the focus of the study from corporate-level governance to Institutional Quality indicators of the country (Porta et al., 1998; Ball et al., 2000; Claessens and Fan, 2002; Hooper et al., 2009). Asongu (2011) analyzed the effect of government policies and institutions on the African stock market and suggested that better Institutional Quality countries would promote bonds with higher market capitalization, better turnover rates, higher stock volume exchanged, and a higher number of companies listed.

Institutional theory suggests that institutional features, rules, regulations, and principles in the exterior environment will restrain the arrangement and behavior of the firms (Meyer and Rowan, 1977; DiMaggio and Powell, 1983). Institutional Quality signifies the institutions’ quality that governs government property rights, laws, traditions, and constitution is crucial for individual relations among the stakeholders (DiMaggio and Powell, 1983). Islam (2020) argued that country-level mechanisms, such as political situation, tax regulation, monetary policy, exchange rate, fiscal policy, and basic infrastructure, also affect the liquidity of the firm. Each country’s regulatory settings are different in various countries, which leads to the different behavior of the firm through the capital market (Clayman et al., 2012).

Scholars have noticed the influence of new terms and the combination of prevailing resources with modest compensations (Grant, 1996). The introduction of the complementary assets concept (resource complementarities) was introduced by Teece (1986). These can be aptitudes or resources from which the firm gets benefits linked with technology, policy, or innovation. Authors recommended that the firm requires complementary resources and a combination of facilities with advantageous conditions to design a new product’s profitability. However, the resource complementary concept is basically recommended for firm-level study (Teece, 1986). Krishnan and Teo (2012) have enlarged its fundamental argument to the country level and have recognized its effectiveness in their empirical study. In line with them, we have used the Institutional Quality index developed *via* PCA composed of six world governance indicators (WGI) as country-level complementary assets that will affect the relationship between corporate governance and stock liquidity.

Based on Weill’s (1992) conversion effectiveness concept, this study postures that Institutional Quality boosts the conversion of resources (corporate governance quality) to production (stock liquidity). This study attempts to the role of Institutional Quality at the country level in improving stock liquidity in an environment characterized by weak corporate governance under the complementary assets view, i.e., intuitional quality strengthens the positive association of the stock liquidity with corporate governance quality. This argument is in line with Weill (1992); term “conversion effectiveness”: Institutional Quality strongly affects how resources (i.e., corporate governance) are efficiently converted to production measures (i.e., stock liquidity). In sum, it is logical to assume that when high Institutional Quality is combined with quality corporate governance, it will enhance stock liquidity. By following the above discussion, we hypothesized that

**H2:** Institutional Quality strengthens the positive relationship between corporate governance and stock liquidity.

## Materials and methods

### Data

The main aim of this study was to analyze the moderating role of Institutional Quality on the relationship between corporate governance and stock liquidity of 230 non-financial companies listed on the PSX for the period of 2009–2019, approximately 11 years. Financial firms are excluded from this study because they vary from non-financial firms in financial structure (Fama and French, 1992).

Secondary data are used for empirical analysis as well as financial data predominantly from the business recorder, the PSX database, and the State Bank of Pakistan (SBP) database for the stock prices, share traded or stock price, and market capitalization. Data related to Institutional Quality are collected from the World Bank portal. Institutional Quality is measured by WGI. Corporate governance data are hand collected from 230 non-financial firms listed on the PSX annual reports. The Table 1 shows measurements and abbreviations of the variables.

**TABLE 1:** Variable descriptions.

Variable	Abbreviation	Measurement
**Dependent variables (stock liquidity)**		
** Price impact frequency**		
Amihud illiquidity estimate	Amihud	Daily ratio of absolute stock return to trading volume in Pakistani rupees averaged over the number of trading days in the financial year.
Liquidity ratio	Amivest stock return in a year	Sum of daily trading volume over the sum of absolute
** Trading frequency**		
Turnover-adjusted zero daily volumes	LM	Turnover-adjusted zero daily volumes
** Trading cost**		
Zero return measure	Zero	Proportion of zero daily returns over number of trading days in the financial year
Independent variables		
The corporate governance index	CG_Index	Composed of the following variables
Board of directors		(1) Board independence.
		(2) CEO duality
		(3) Board size
		(4) Board meeting
		(5) Gender diversity
Audit committee		(1) Audit committee size
		(2) Audit committee meeting
		(3) Audit committee independence
Ownership concentration	Top_Own	(1) Shares of largest shareholder divided by total number of outstanding shares
Institutional ownership	Inst_Own	(1) Shares owned by institutions divided by total number of outstanding shares
**Moderator**		
Institutional Quality	IQ_Index	Political stability, rule of law, regulatory quality, control of corruption, government effectiveness, and voice and accountability
**Control variables**		
Firm size	Size	Number of outstanding shares times share price at the end of fiscal year
Leverage	Leverage	Book value of total liabilities over book value of total assets
Firm age	Age	The year, firm listed at the PSX
Stock price	S_Price	Natural log of stock price
Volatility	VOLATILITY	Daily stock return’s SD

Source: Author’s calculations (2020).

### Why Pakistan?

The main motivation of this study is to analyze if the Institutional Quality moderates the relationship between corporate governance and stock liquidity. This topic is not yet addressed in the finance literature to the best of our knowledge. It is essential to understand how corporate strategies affect the microstructure, and if this relationship can be moderated by Institutional Quality, as this can help monitors to design relevant trade regulations and help shareholders and investors to set comprehensive strategies for their stock trading.

Studies by Chung et al. (2010) and Ahmed and Ali (2017) directly analyzed the relationship between corporate governance and stock liquidity based solely on developed markets in the United States and Australia. Due to regulatory and institutional differences, it is not clear whether their results can be generalized to countries in which the market is not developed, and it has not yet focused on Institutional Quality. Emerging markets such as Pakistan represent a significant alternate setting to analyze this problem for multiple reasons.

First, Institutional Quality is measured by an independent index composed of six WGI. According to the transparency international report 2021, Pakistan’s current corruption index rank is so high and is at 140th position as compared to other developed or developing countries. We are having a lake of political stability that from the day of independence till now, none of the prime ministers has completed his tenure. Recently on 03 March 2022, the president of Pakistan dissolved the national assembly of Pakistan again. Furthermore, Pakistan has the lake of government effectiveness as well as the rule of law. Second, Pakistan has highly concentrated family ownership. The corporate boards of such organizations act as a rubber stamp, and one family holds the bulk of the shares. Such companies are owned by individuals, the state, and international executives, and these stakeholders actively participate in the companies’ affairs and weaken the objectivity and discretion of the board.

Third, Pakistani firms rely mostly on loans from banks as a major source of financing; thus, capital market financing plays a more passive role than in developed markets. As Pakistani firms rely much on capital market financing, stock liquidity also plays a different role in Pakistan. Fourth, the public capital market is not developed as in the United States and displays weak financial transparency. This causes information asymmetry and problems of adverse selection, resulting in a significantly less liquid market. More specifically, Pakistan’s financial markets are not sophisticated and have yet to gain the level of information transparency found in the developed markets. Its financial analysts do not provide the same level of information to investors, making it difficult for Pakistani investors to depend on information disclosed directly by firms.

An information environment depends on the quality of corporate governance (Leuz et al., 2003; Chung et al., 2010). The importance of corporate governance is made clear by introducing the first corporate governance code by the Security and Exchange Commission of Pakistan (SECP) in March 2002, which was subsequently revised in 2013 and 2017. The role of corporate governance in increasing transparency and enhancing stock liquidity is even more critical in Pakistan than in developed economies. Due to these characteristics, Pakistan provides an ideal setting to analyze the effect of corporate governance on stock liquidity and the moderating role of Institutional Quality.

### Variable measurement

#### Dependent variable (stock liquidity)

Stock liquidity is used as a dependent variable in this study. In the financial field, liquidity is very critical. We have used four stock liquidity measures, i.e., Amihud Illiquidity Estimate, Zero Return Measure, Liquidity Ratio (AMIVEST), and Turnover-Adjusted Zero Daily Volume.

##### Zero return measure

“Zero-return measure” (also known as “null-return estimate”) is the number of zero daily return days reported in a year. Lesmond et al. (1999) explained that the zero-return measure is positively associated with spreading measures, consistent with the cost-effectiveness of purchases on inventory returns. The following formula calculates this measure:


(1) $${\mathrm{Zero}}_{\mathrm{it}=}\,\frac{{\mathrm{ZR}}_{\mathrm{it}}}{{\mathrm{TD}}_{\mathrm{it}}}$$

where *ZR*_*it*_ is the number of zero-return day in year *t* for firm *i*, and *TD*_*it*_ is the number of trade days in year *t* for firm *i*. A higher value indicates lower stock liquidity.

##### Amihud illiquidity estimate

The certain return on trading in Pakistani rupees (Amihud illiquidity estimate, ILLIQ) is measured as the total stock return collected on numerous trade days through the financial year. It measures the extent to which the actual stock price varies with the volume of trading, calculated as follows:


(2) $$\mathrm{ILLI}Q_{\mathrm{it}}={\frac{1}{D}}_{t}\sum_{d=1}^{D_{\mathrm{iy}}}\frac{\left|R_{\mathrm{itd}}\right|}{\mathrm{VOL}D_{\mathrm{itd}}}$$

where *idt* stands for the absolute stock return of firm i for the year *t*, *VOLD*_*idt*_ is the volume of firm *i* on the *d* of year *t*, and *D*_*iy*_ is the number of days available for company *i* on the *d* of year *t*. As *ILLIQ* rises, stock liquidity decreases.

##### Liquidity ratio (AMIVEST)

The liquidity ratio (AMIVEST) is calculated as the volume of trading associated with a stock price change unit used in a number of studies (Amihud et al., 1997; Berkman and Eleswarapu, 1998). Datar et al. (1998) calculated the liquidity ratio as follows:


(3) $${\mathrm{AMIVEST}}_{\mathrm{it}=}\,\sum_{t}{\mathrm{VOL}}_{\mathrm{it},/}\,\sum_{t}\left|R_{\mathrm{it}}\right|$$

where the limit is exchanged and where the average total stock returns are, respectively, for *VOL*_*it*_ and in the year *t*.

##### Turnover-adjusted zero daily volume

Liu (2006) suggested a new measure of stock liquidity, namely, the sales-adjusted zero daily volume (LM). LM focuses on the trading speed; however, it does capture several liquidity dimensions. It is measured as follows:


(4) $${\mathrm{LM}}_{\mathrm{it}=}\,\left[{\mathrm{NoZV}}_{\mathrm{it}}+\frac{1/\left(\mathrm{turn}\,{\mathrm{over}}_{\mathrm{it}}\right)}{\mathrm{Deflator}}\right]\times \frac{252}{{\mathrm{NoTD}}_{\mathrm{it}}}$$

where *NoZV*_*it*_ is the number of zero-day volumes for the company *i* in the year *t*; turnover (T) is the inventory of company *i* in year *t*; *NoTD*_*t*_ is the total number of days of trading in the year *t*; and deflators are set at 480,000 (Liu, 2006). The NoTD element multiplication *t* standardization makes LM equal over time, thus standardizing trading days within 1 year. A more excellent LM value indicates lower liquidity.

#### Corporate governance (independent variable)

The CGI is an independent index of governance mechanisms developed through PCA consisting of the following components, i.e., board independence, board size, board meetings, board diversity, CEO duality, ownership concentration, institutional ownership, audit independence, audit size, and audit meetings.

#### Institutional Quality (moderator)

The study used Institutional Quality index developed *via* PCA composed of WGI indicators, i.e., Political stability, control of corruption, regulatory quality, the rule of law, voice and accountability, and government effectiveness (Easterly, 2002; Al-Marhubi, 2004; Méon and Weill, 2005; Bjørnskov, 2006; Kaufmann et al., 2009; Langbein and Knack, 2010).

### Research model

We estimated the following baseline models to test whether the Institutional Quality moderates the relationship between corporate governance and stock liquidity and whether corporate governance quality impacts stock liquidity.

#### Corporate governance and stock liquidity

To analyze H1, we have used the following model, where SL stands for stock liquidity measured *via* Amihud, Amivest, Zero, and LM.


(5) $${\mathrm{SL}}_{\mathrm{it}=\beta_{0}+\beta_{1}\,{\mathrm{CGI}}_{\mathrm{it}}+\mathrm{CONTROLS}+\epsilon_{\mathrm{it}}}$$

Corporate governance index is used as a variable of interest developed *via* PCA; according to Ali et al. (2017), we used control variables, such as firm size and leverage and, according to Biswas (2020), firm age, stock price, and volatility.

#### Corporate governance, Institutional Quality, and stock liquidity


(6) $${\mathrm{SL}}_{\mathrm{it}=\beta_{0}+\beta_{1}\,{\mathrm{CGI}}_{\mathrm{it}}+\beta_{2}\,{\mathrm{IQ}}_{\mathrm{it}}+\beta_{3}\,{\mathrm{IQ}}_{\mathrm{it}}\times {\mathrm{CGI}}_{\mathrm{it}}+\mathrm{CONTROLS}+\epsilon_{\mathrm{it}}}$$

To test H2, we have used the subsequent model, where SL stands for stock liquidity measured *via* Amihud, Amivest, Zero, and LM. We have also used Institutional Quality index (IQ) composed of WGI, i.e., political stability, rule of law, regulatory quality, control of corruption, government effectiveness, and voice and accountability. By following Ali et al. (2017), we have used control variables, such as firm size and leverage, and by following Biswas (2020), firm age, stock price, and volatility.

## Results and discussion

### Descriptive statistics

This Table 2 shows the descriptive statistics for all the measures of stock liquidity, i.e., Amihud illiquidity estimate (Amihud), Liquidity ratio (Amivest), Zero-return measure (Zero), and Turnover adjusted zero daily volume (LM). Also, for independent variables, the moderator Institutional Quality index and control variables for the sample period of 2009–2019.

**TABLE 2:** Descriptive statistics.

Variables	Observations	Mean	SD	Min	Max
Amihud	2,485	0.002	0.009	1.11e−09	0.189
Zero	2,485	0.100	0.133	0	0.944
Amivest	2,392	767.994	3,669.432	0	6,3920.07
LM	2,423	17.83	157.3	1.11e−07	297
B_Size	2,465	2.066	0.166	1.609	3.045
B_Indepeendce	2,465	0.175	0.188	0	1
B_Meeting	2,407	1.639	0.316	0	3.497
B_Diversity	2,465	0.0945	0.139	0	1
CEO_Duality	2,466	0.172	0.377	0	1
Audit_Size	2,463	1.195	0.179	0.693	2.079
Audit_Meeting	2,428	1.421	0.124	0	2.485
Inst_Own	2,462	0.106	0.128	0	0.895
Inst_Own	2,462	0.106	0.128	0	0.895
Top5_Own	2,463	0.657	0.208	0	0.999
CG_Index	2,389	1.15e−08	1.000	−2.747	5.886
IQ_Index	2,466	1.10e−09	1.000	−1.556	1.521
Leverage	2,456	0.598	0.329	0.00433	3.146
Size	2,440	21.338	2.344	0	30.612
Age	2,466	43.75	18.17	13	160
S_Price	2,443	3.745	1.860	−4.605	9.350
VOLATILITY	2,489	0.0518	0.0616	0.00855	0.775

Source: Author’s calculation (2020).

Amihud is calculated as the ratio of daily absolute stock return to volume in Pakistani rupees averaged over a number of trading days in the financial year. The mean value for Amihud is 0.00153 followed by a SD value of 0.00868. Amihud minimum value is 1.11e−09 to the maximum value of 0.189 for 2009–2019. The mean value of the liquidity ratio (Amivest) is 1.0050 with a SD of 9.99100. Amivest ranges from the minimum value of 0 to the maximum of 4.36300. The moderator of the study is Institutional Quality index, which has a mean value of 1.10e−09 that ranges from a minimum value of −2.747 to a maximum value of 1.521 with a SD of 1.000.

### Corporate governance and stock liquidity

#### Corporate governance index

We have used PCA to develop a CGI. The main objective of PCA is to decrease the number of variables in uncorrelated mechanisms. There are certain advantages of incorporating PCA, i.e., it enables us to integrate information about the specific set of corporate governance appliances into a solo index (Agrawal and Knoeber, 1996). Besides this, PCA can be a regulator for the likely presence of multicollinearity between the distinct corporate governance variables (Bebchuk and Cohen, 2005). PCA allocates weights to dissimilar variables spontaneously, slightly more than allocating these weights randomly or parallel. We have to address two problems before determining the rationality of PCA. First, the correlations among variables must be high than that among errors (sample adequacy). Second, the correlation matrix must be factorable, i.e., the correlation matrix must be diverse from the individuality matrix (Pett et al., 2003). Incorporating the largest variance of data is the first component of PCA. I have selected the first largest variance for the representation of board independence, the board size, board meetings, board diversity, CEO duality, audit size, audit independence, and audit meetings, as suggested by Tarchouna et al. (2017).

Table 3 shows the weights of all variables of the CG index, which is developed through PCA. It depicts that the contribution of board size, board independence, and board meetings are positive to the index. It means that the board with more independent directors will have good monitoring control and such firms will have strong governance. Similarly, the board meeting’s positive contribution to the index means that frequent meetings will reduce the information asymmetry and agency conflicts as well for Pakistani firms. CEO duality and board diversity have a negative contribution to the board index. Audit committee size and audit independence, and audit meetings have positive contribution to the index.

**TABLE 3:** Corporate governance index.

Variables	Weights	KMO
B_Size	0.5313	0.6007
B_Independence	0.2608	0.5829
B_Meeting	0.2382	0.5961
B_Diversity	−0.2317	0.6762
CEO_Duality	−0.3080	0.6385
Audit_Size	0.5306	0.5971
Audit_Indep	0.2839	0.5624
Audit_Meeting	0.1932	0.5833
Top5_Own	−0.0138	0.4530
Inst_Own	0.2114	0.5992
Kaiser–Meyer–Olkin statistic		0.597
Bartlett’s Chi-square		1,414.720
Bartlett’s test *p*-value		0.000

Source: Author’s calculation (2020).

Raheja (2005) suggested that firms with an independent audit have good corporate governance. It also shows that the contribution of top five ownership and institutional ownership is negative to the index. Before reporting the index, two concerns have been addressed as suggested by Tarchouna et al. (2017). To ensure the correlation among variables is higher than the correlation among the errors, we have used the Kaiser–Meyer–Olkin (KMO) test, and its value is 0.6007, and to ensure that variables are factorable, we have used Bartlett’s test for sphericity (*p*-value < 0.001).

#### Corporate governance and stock liquidity (OLS regression)

In Table 4, we have regressed stock liquidity proxies, i.e., Amihud illiquidity estimate (Amihud) and turn over adjusted zero daily volumes (LM) with the corporate governance index CG_Index developed through PCA. Table 4 shows a negative association between the CGI and the stock liquidity measure (Amihud), significant at 5%, which states that a decrease in Amihud leads to an increase in stock liquidity. After controlling for industry fixed effects, we found the same results for CG_Index and Amihud. Our results support hypothesis H1 of the study, and our results are parallel with those of Ali et al. (2017).

**TABLE 4:** Corporate governance and stock liquidity (OLS).

Variables	Amihud	Amihud	LM	LM
CG_Index	−0.000**	−0.000**	−0.849*	−0.076**
	(−2.362)	(−2.007)	(−1.650)	(−0.132)
Leverage	−0.002	−0.001	5.623***	7.394***
	(−1.579)	(−1.591)	(2.660)	(3.332)
Size	−0.000195*	−0.000203	−5.094***	−5.150***
	(−1.870)	(−1.581)	(−10.58)	(−9.039)
Age	0.000	0.0001	1.161	2.527*
	(1.175)	(1.313)	(0.986)	(1.677)
S_Price	−6.70e−05	−0.000130	4.788***	6.091***
	(−0.633)	(−0.904)	(8.766)	(8.801)
Volatility	0.0796***	0.0788***	64.32***	56.60***
	(4.721)	(4.620)	(4.179)	(3.753)
Constant	0.001	0.001	91.83***	71.65***
	(0.449)	(0.268)	(8.617)	(5.982)
Observations	2,332	2,332	2,321	2,321
R-squared	0.318	0.325	0.113	0.159
Industry FE	No	Yes	No	Yes

Robust t-statistics in parentheses.

***p < 0.01, **p < 0.05, *p < 0.1.

#### Corporate governance and stock liquidity (two-stage least squares estimation)

We have used a two-stage least squares (2SLS) method to address the issue of reverse causality. This approach demands an IV, which is highly correlated with the endogenous variable (CG) but does not have a direct effect on the dependent variable (stock liquidity) (Kennedy, 2003). The table shows the 2SLS results. In the first stage, we have regressed the CGI, which is a self-developed index *via* PCA, including 11 governance measures.

Following the studies by Jiraporn et al. (2011); Liu et al. (2014), and Liu et al. (2015), we considered the first IV Indus_CG_Index as the Industrial governance index, which is calculated as (industry governance index − firm governance level index / total observation in the industry − 1). The perception behind considering industrial corporate governance as an IV is that provisions of a firm’s governance (such as board and its subcommittees) may be strongly correlated with the industry peers due to similar corporate assortment and investment prospects, but such industrial governance is improbable to affect stock liquidity directly (Yang and Zhao, 2014). By following Prommin et al. (2014) and Jiraporn et al. (2015), we used the second IV, the corporate governance act 2013 (CG_Act), which is a binary variable equal to 1 for the year after 2013 and 0 before 2013. The use of the CG act as an IV is based on the notion that after the corporate governance act, the period of 2013–2019, firm-level CG should be advanced, proposing that the firm’s CG and (CG_Act) are highly correlated. However, the CG_Act must affect stock liquidity only through firm CG. Table 5 shows significant negative relation of CG_Index and Amihud at 1%, which states that good governance will decrease Amihud, which leads to an increase in stock liquidity. The table shows negative relation between CG_Index and LM, which is significant at 5%. Our results are parallel to those of Ali et al. (2017) and are not endogenous. Our results affirm hypothesis H1 of the study that good corporate governance will enhance stock liquidity.

**TABLE 5:** Corporate governance and stock liquidity (2SLS).

Variables	First stage CG_Index	Second stage Amihud	Second stage LM
CG_Index		−0.005***	−5.108**
		(−3.705)	(−1.049)
Indus_CG_Index	0.237***		
	(6.031)		
CG_Act	−0.121***		
	(−2.924)		
Leverage	0.205***	−0.002***	6.590***
	(3.070)	(−3.190)	(2.623)
Size	0.178***	−0.001***	−4.298***
	(13.89)	(−3.930)	(−4.012)
Age	0.429***	−0.001**	3.505
	(8.693)	(−2.163)	(1.316)
S_Price	−0.0636***	0.000	4.472***
	(−3.897)	(1.529)	(6.752)
Volatility	−0.426	0.082***	61.77***
	(−1.241)	(24.90)	(5.183)
Constant	−5.170***	0.028***	67.09**
	(−15.96)	(3.419)	(2.238)
Observations	2,302	2,302	2,291
R-squared		0.077	0.100
Industry FE	No	No	No

Robust t-statistics in parentheses.

***p < 0.01, **p < 0.05, *p < 0.1.

### Corporate governance, Institutional Quality, and stock liquidity

#### Institutional Quality index

The Institutional Quality index is developed *via* PCA composed of six WGI, i.e., control of corruption, government efficiency, political stability, regulatory quality, the rule of law, and voice and accountability. These variables contribute positively to the Institutional Quality index.

Before reporting the index, two concerns have been addressed, as suggested by Tarchouna et al. (2017). To ensure that the correlation among variables is higher than the correlation among the errors, we have used the KMO test, and its value is 0.511 as reported in Table 6; to ensure that variables are factorable, we have used the Bartlett’s test for sphericity (*p*-value < 0.001).

**TABLE 6:** Institutional Quality index.

Variables	Weights
Control of corruption	0.5989
Government effectiveness	0.2086
Political stability	0.5596
Regulatory quality	0.1244
Rule of law	0.4957
Voice and accountability	0.1530
Kaiser–Meyer–Olkin statistic	0.511
Bartlett’s Chi-square	6598.078
Bartlett’s test *p*-value	0.000

Source: Author’s Calculation (2020).

#### Corporate governance, Institutional Quality, and stock liquidity (OLS regression)

In Table 7, we have regressed stock liquidity either as Amihud and LM with interaction terms of CG_Index and Institutional Quality index developed *via* PCA including the control of corruption, government efficiency, political stability, regulatory quality, rule of law, voice, and accountability. We also controlled for firm size, leverage, firm age, stock price, volatility, and industry fixed effects. The table shows positive relationship between Amihud and interaction terms of CG_Index and IQ_Index and is significant at 1%, which states that the strong Institutional Quality in a year will boost the relationship between corporate governance and stock liquidity.

**TABLE 7:** Corporate governance, Institutional Quality, and stock liquidity (OLS).

Variables	Amihud	Amihud	LM	LM
CG_Index × IQP_Index	0.001***	0.001***	9.377**	12.13*
	(3.834)	(3.856)	(2.487)	(1.940)
CG_Index	0.001	0.001	−0.948*	−0.136
	(1.465)	(1.586)	(−1.831)	(−0.238)
IQP_Index	−0.002***	−0.002***	−1.519**	−2.055***
	(−9.247)	(−9.090)	(−2.423)	(−3.308)
Leverage	−0.001	−0.001	5.898***	7.876***
	(−1.216)	(−1.141)	(2.804)	(3.564)
Size	−6.49e−05	2.39e−05	−4.973***	−4.870***
	(−0.609)	(0.177)	(−9.958)	(−8.089)
Age	0.000224	0.001	0.996	2.332
	(0.684)	(0.979)	(0.844)	(1.552)
S_Price	7.80e−05	3.17e−05	4.929***	6.302***
	(0.749)	(0.227)	(9.197)	(9.252)
Volatility	0.0813***	0.081***	66.07***	59.41***
	(4.845)	(4.754)	(4.279)	(3.931)
Constant	−0.00189	−0.005	89.06***	64.58***
	(−0.725)	(−1.536)	(8.078)	(5.045)
Observations	2,332	2,332	2,321	2,321
R-squared	0.351	0.358	0.115	0.162
Industry FE	No	Yes	No	Yes

Robust t-statistics in parentheses.

***p < 0.01, **p < 0.05, *p < 0.1.

And after controlling for industry fixed effects, we found the same results. The third column of the table shows a positive and significant coefficient for LM and interaction term of CG_Index and IQ_Index, which states that the relationship between corporate governance and stock liquidity is positively moderated by the Institutional Quality, and we found the same results for this relationship after controlling the industry fixed effect which is significant at 1%. Our results support the hypothesis of the study that Institutional Quality positively moderates the relationship between corporate governance and stock liquidity.

#### Corporate governance, Institutional Quality, and stock liquidity (two-stage least squares estimation)

The 2SLS analysis is an alternate way to deal with potential endogeneity. This approach includes IVs that is highly correlated with CGI but not with liquidity. By following Jiraporn et al. (2015) and Yang and Zhao (2014), we have used an IV, Indus_CG_Index, as the industrial governance index, which is calculated as (industry governance index − firm governance level index / total observation in industry − 1).

The second IV is the interaction term between Indus_CG_Index and IQP_Index. The intuition behind using the interaction term of Indus_CG and IQP_Index as an IV is that the endogenous variable CG is included individually in the basic model as well as in the interaction term. The table shows dependent variables in the second stage that Amihud illiquidity estimate (Amihud) and turn over adjusted zero daily volumes (LM). We have also control for leverage, firm size, firm age, stock price, and volatility.

Table 8 shows the 2SLS results. In the first stage, we have regressed the CGI, which is a self-developed index *via* PCA, including 11 governance measures. The table depicts the IV’s positive significant at 1% and the second variable’s negative significant at 5%, stating that IVs are not weak. The table shows that the interaction term of CG_Index and IQ_Index is positively significant with stock liquidity (Amihud). In the end, Table 8 depicts a significant positive relationship between the interaction terms of CG_Index and IQ_Index with stock liquidity (LM). Our results show that there is no problem with endogeneity, and the results support hypothesis H2 of the study that Institutional Quality positively moderates the relationship between corporate governance and stock liquidity.

**TABLE 8:** Corporate governance, Institutional Quality, and stock liquidity (2SLS).

	First stage	Second stage	Second stage

Variables	CG_Index	Amihud	LM
CG_Index × IQP_Index		0.003***	7.121**
		(4.161)	(2.538)
CG_Index		0.0010	−13.49**
		(0.642)	(−1.998)
Indus_CG_Index	0.229***		
	(5.765)		
Indus_CG_Index × IQP_Index	−0.081**		
	(−2.269)		
IQP_Index	−0.04**	−0.002***	−2.173***
	(−2.366)	(−8.413)	(−2.720)
Leverage	0.199***	−0.001	8.843***
	(2.983)	(−1.383)	(3.141)
Size	0.177***	−0.0002	−2.436*
	(13.84)	(−0.503)	(−1.720)
Age	0.431***	0.000	6.799**
	(8.758)	(0.158)	(2.116)
S_Price	−0.065***	2.14e−05	4.034***
	(−4.050)	(0.128)	(5.606)
Volatility	−0.339	0.079***	59.89***
	(−0.982)	(26.22)	(4.685)
Constant	−5.232***	0.001	15.43
	(−16.07)	(0.110)	(0.389)
Observations	2,302	2,302	2,291
R-squared		0.256	−0.009
Industry FE	No	No	No

*t*-statistics in parentheses.

***p < 0.01, **p < 0.05, *p < 0.1.

### Robustness checks

For robustness, we used alternative proxies to measure stock liquidity (i.e., Amivest and zero); these measures are widely used in the literature. These two proxies are commonly used in previous literature. We have used these measures for robustness. Our model is estimated by these measures using 2SLS method, as reported in the table. The coefficients reported in Table 9 depict that the results did not change with alternative proxies of stock liquidity. The results are significantly negative, consistent with the previous results.

**TABLE 9:** Robust check of CG_Index and stock liquidity (2SLS).

	First stage	Second stage	Second stage

Variables	CG_Index	Zero	Amivest
CG_Index		−0.0392*	5.100e+08**
		(−1.915)	(0.886)
Indus_CG_Index	0.237***		
	(6.031)		
CG_Act	−0.121***		
	(−2.924)		
Leverage	0.205***	−0.00616	6.991e+08**
	(3.070)	(−0.585)	(2.404)
size	0.178***	−0.0377***	8.279e+08***
	(13.89)	(−8.385)	(6.593)
age	0.429***	−0.00233	1.207e+08
	(8.693)	(−0.208)	(0.382)
S_Price	−0.0636***	0.0179***	−6.802e+08***
	(−3.897)	(6.460)	(−8.805)
Volatility	−0.426	−0.118**	−1.251e+09
	(−1.241)	(−2.353)	(−0.910)
Constant	−5.170***	0.857***	−1.516e+10***
	(−15.96)	(6.802)	(−4.296)
Observations	2,302	2,302	2,281
R-squared		0.065	0.083
Industry FE	No	No	No

*t*-statistics in parentheses.

***p < 0.01, **p < 0.05, *p < 0.1.

The results are robust to explain the positive moderating role of Institutional Quality on corporate governance and stock liquidity as reported in Table 10. And the results of corporate governance and stock liquidity are also significant and robust with the alternative proxies of stock liquidity. Our results are in line with the literature (Ali et al., 2017) on corporate governance and stock liquidity.

**TABLE 10:** Robust check of CG_Index, IQ_Index, and stock liquidity.

	First stage	Second stage	Second stage

Variables	CG_Index	Zero	Amivest
CG_Index × IQP_Index		0.033**	5.708e+08*
		(2.329)	(1.691)
CG_Index		−0.101***	6.119e+08
		(−3.265)	(0.824)
Indus_CG_Index	0.229***		
	(5.765)		
Indus_CG_Index × IQP_Index	−0.0808**		
	(−2.269)		
IQP_Index	−0.0465**	−0.037***	2.733e+08***
	(−2.366)	(−10.00)	(3.159)
Leverage	0.199***	0.032**	4.480e+08
	(2.983)	(2.533)	(1.478)
Size	0.177***	−0.006	5.996e+08***
	(13.84)	(−0.930)	(3.879)
Age	0.431***	0.054***	−2.679e+08
	(8.758)	(3.729)	(−0.755)
S_Price	−0.0658***	0.009***	−6.460e+08***
	(−4.050)	(3.001)	(−8.273)
Volatility	−0.339	−0.178***	−1.836e+09
	(−0.982)	(−3.057)	(−1.332)
Constant	−5.232***	−0.0224	−8.791e+09**
	(−16.07)	(−0.124)	(−2.022)
Observations	2,302	2,302	2,281
R-squared		−0.234	0.106
Industry FE	No	No	No

*t*-statistics in parentheses.

***p < 0.01, **p < 0.05, *p < 0.1.

## Conclusion

This study empirically analyzes the moderating effect of Institutional Quality on the relationship between corporate governance and stock liquidity for non-financial firms listed on the PSX. The study uses a sample of 230 non-financial firms listed on the PSX during the time period of 2009–2019 to analyze whether corporate governance practices affect stock liquidity in Pakistan. Whether Institutional Quality moderates the relationship between corporate governance and stock liquidity is still unclear. We provided analytical proof of stock liquidity in the context of information asymmetry and agency theory.

To the best of our knowledge, this is the first study in the field of finance to analyze the moderating role of Institutional Quality on the relationship between corporate governance and stock liquidity. This is also the first study to develop a new index for Institutional Quality and corporate governance *via* PCA. This study further contributes to the existing literature and policy in different ways. We have tried to fill this void and give a comprehensive picture of stock liquidity in the previous literature. This is the first research for the PSX to shed light on the issue through 230 non-financial companies.

The emerging market corporate governance structure differs from the established markets in rising economies like Pakistan, where most firms are owned and monitored by family members and managers. Also, between the marginal investors and the administration (the governing family), there is a big agency problem. These companies are normal in their corporate governance quality in a different legal setting, business, and institutional facilities than in developed countries. This study is therefore an attempt to overcome this void. The results of the study show how Institutional Quality moderates the relationship between corporate governance and stock liquidity and at which point it influences Pakistani stock markets and contributes significantly to Pakistani stock market literature.

The findings of the study suggest a highly significant, positive moderating role of Institutional Quality in the relationship between corporate governance and stock liquidity. It means that an increase in the country’s government efficiency, political stability, control of corruption, and rule of law will boost corporate governance, which will lead to an increase in stock liquidity, as the whole improvement in the Institutional Quality index positively moderates the relationship between corporate governance and stock liquidity, which is in line with hypothesis 2 of the study.

The findings regarding the impact of corporate governance on stock liquidity in Pakistani firms were found to be highly significant and positive. As Pakistan has weak corporate governance, this research will help the sector to improve governance to enhance stock liquidity. The study observed a positive and significant impact of corporate governance on stock liquidity; the results are consistent with agency and information asymmetry theory. The results are robust *via* alternative proxies of stock liquidity. Our results are in line with the previous literature on corporate governance and stock liquidity.

The findings of this study can be applied to such emerging economies where the stock market is not developed and has weak corporate governance. This can also be applied to emerging markets where ownership is highly concentrated and mostly family ownership like Pakistan. Furthermore, the results of this study can also be applicable to such emerging countries lakes political stability, high corruption index and the lake rule of law. The results of this study have significant implications. The findings of this study will help regulators to formulate policy protocols that enhance stock liquidity. Additionally, this finding might help traders and investors plan their trading approaches by considering the corporate governance mechanisms of this research closely. These results also have administrative implications, as listed firms may adopt the best Institutional Quality to enhance stock liquidity that improves information asymmetry between share traders.

### Limitations and future research directions

The long time period will permit the study to explain major events like financial crises and the introduction of the first corporate governance code. This study analyzes data from only one developing country. However, considering cultural and legal distinctions in a story, the results can be applied to other developing economies. This study can also be performed on an international sample using data from multiple countries to analyze the impact of individual corporate governance channels on stock liquidity. Future research could study the impact of shareholder protection and disclosure quality on stock liquidity. The findings of this study encourage the proposal that corporations, managers, and investors be harsher in the supervision of corporate governance structures, with the aim of enlisting trade laws and developing the corporate atmosphere and trading system. In addition, the study focuses on the fundamental role of audit committee independence in market liquidity. It is critical to evaluate the value of this variable by precisely recognizing the independent non-executive board directors in the Corporate Governance Code, and the regulators pay specific attention to this information.

## Data availability statement

The raw data supporting the conclusions of this article will be made available by the authors, without undue reservation.

## Author contributions

SA presented the idea and analysis and wrote the manuscript. WZ and GF supervised and reviewed the article. ZA helped sort out data, helped in methodology, and reviewed the article. MC helped in write-up. All authors contributed to the article and approved the submitted version.

## References

[B1] Agostino M. Di Tommaso M. R. Nifo A. Rubini L. Trivieri F. (2020). Institutional quality and firms’ productivity in European regions. *Reg. Stud.* 54 1275–1288. 10.1080/00343404.2020.1712689

[B2] Agrawal A. Knoeber C. R. (1996). Firm performance and mechanisms to control agency problems between managers and shareholders. *J. Financ. Quant. Anal.* 31 377–397. 10.2307/2331397

[B3] Ahmed A. Ali S. (2017). Boardroom gender diversity and stock liquidity: Evidence from Australia. *J. Contemp. Account. Econ.* 13 148–165. 10.1016/j.jcae.2017.06.001

[B4] Ajina A. Lakhal F. Sougné D. (2015). Institutional investors, information asymmetry and stock market liquidity in France. *Int. J. Manag. Finance* 11 44–59. 10.1108/IJMF-08-2013-0086

[B5] Ali S. Liu B. Su J. J. (2017). Corporate governance and stock liquidity dimensions: Panel evidence from pure order-driven Australian market. *Int. Rev. Econ. Finance* 50 275–304. 10.1016/j.iref.2017.03.005

[B6] Al-Marhubi F. (2004). The determinants of governance: A cross-country analysis. *Contemp. Econ. Policy* 22 394–406. 10.1093/cep/byh029

[B7] Amihud Y. Mendelson H. (1986). Liquidity and stock returns. *Financ. Anal. J.* 42 43–48. 10.2469/faj.v42.n3.43

[B8] Amihud Y. Mendelson H. (2006). Stock and bond liquidity and its effect on prices and financial policies. *Financ. Mark. Portf. Manag.* 20 19–32. 10.1007/s11408-006-0001-y

[B9] Amihud Y. Mendelson H. Lauterbach B. (1997). Market microstructure and securities values: Evidence from the Tel Aviv Stock Exchange. *J. Financ. Econ.* 45 365–390. 10.1016/S0304-405X(97)00021-4

[B10] Andrianova S. Demetriades P. Xu C. (2011). Political economy origins of financial markets in Europe and Asia. *World Dev.* 39 686–699. 10.1111/j.1747-7379.2011.00847.x 22069767

[B11] Asongu S. A. (2011). Why Do French Civil–Law Countries Have Higher Levels Of Financial Efficiency? *J. Adv. Res. Law Econ.* 2 94–108.

[B12] Bacidore J. M. Sofianos G. (2002). Liquidity provision and specialist trading in NYSE-listed non-US stocks. *J. Financ. Econ.* 63 133–158. 10.1016/S0304-405X(01)00092-7

[B13] Ball R. Kothari S. Robin A. (2000). The effect of international institutional factors on properties of accounting earnings. *J. Account. Econ.* 29 1–51. 10.1016/S0165-4101(00)00012-4

[B14] Bebchuk L. A. Cohen A. (2005). The costs of entrenched boards. *J. Financ. Econ.* 78 409–433. 10.1016/j.jfineco.2004.12.006

[B15] Berkman H. Eleswarapu V. R. (1998). Short-term traders and liquidity: A test using Bombay Stock Exchange data. *J. Financ. Econ.* 47 339–355. 10.1016/S0304-405X(97)00048-2

[B16] Biswas P. K. (2020). Corporate governance and stock liquidity: Evidence from a speculative market. *Account. Res. J.* 33 323–341. 10.1108/ARJ-01-2019-0005

[B17] Bjørnskov C. (2006). The multiple facets of social capital. *Eur. J. Political Econ.* 22 22–40. 10.3389/fpsyg.2022.885616 35936339 PMC9346444

[B18] Brockman P. Chung D. Y. (2003). Investor protection and firm liquidity. *J. Finance* 58 921–937. 10.1111/1540-6261.00551

[B19] Brockman P. Chung D. Y. (2008a). Commonality under market stress: Evidence from an order-driven market. *Int. Rev. Econ. Finance* 17 179–196. 10.1016/j.iref.2007.06.002

[B20] Brockman P. Chung D. Y. (2008b). Investor protection, adverse selection, and the probability of informed trading. *Rev. Quant. Finance Account.* 30 111–131. 10.1007/s11156-007-0049-4

[B21] Çam İ Özer G. (2022). The influence of country governance on the capital structure and investment financing decisions of firms: An international investigation. *Borsa Istanb. Rev.* 22 257–271. 10.1016/j.bir.2021.04.008

[B22] Chen W. P. Chung H. Lee C. Liao W. L. (2007). Corporate governance and equity liquidity: Analysis of S&P transparency and disclosure rankings. *Corp. Gov.* 15 644–660. 10.1016/j.heliyon.2019.e02050 31372533 PMC6658730

[B23] Chung K. H. Elder J. Kim J.-C. (2010). Corporate governance and liquidity. *J. Financ. Quant. Anal.* 45 265–291. 10.1017/S0022109010000104

[B24] Claessens S. Fan J. P. (2002). Corporate governance in Asia: A survey. *Int. Rev. Finance* 3 71–103. 10.1111/1468-2443.00034

[B25] Clayman M. R. Fridson M. S. Troughton G. H. (2012). *Corporate finance: A practical approach.* Hoboken, NJ: John Wiley & Sons.

[B26] Coffee J. C. (1991). Liquidity versus control: The institutional investor as corporate monitor. *Columbia law Rev.* 91 1277–1368. 10.2307/1123064

[B27] Cornell B. Sirri E. R. (1992). The reaction of investors and stock prices to insider trading. *J. Finance* 47 1031–1059. 10.1111/j.1540-6261.1992.tb04004.x

[B28] Daadaa W. (2021). Bid-ask spread, corporate board and stock liquidity in emergent markets. *Afr. J. Econ. Manag. Stud.* 12 531–542. 10.1108/AJEMS-04-2021-0175

[B29] Datar V. T. Naik N. Y. Radcliffe R. (1998). Liquidity and stock returns: An alternative test. *J. Financ. Mark.* 1 203–219. 10.1016/S1386-4181(97)00004-9

[B30] DiMaggio P. J. Powell W. W. (1983). The iron cage revisited: Institutional isomorphism and collective rationality in organizational fields. *Am. Sociol. Rev.* 48 147–160. 10.2307/2095101

[B31] Easterly W. (2002). The cartel of good intentions: The problem of bureaucracy in foreign aid. *J. Policy Reform* 5 223–250. 10.1080/1384128032000096823

[B32] Fama E. F. French K. R. (1992). The cross-section of expected stock returns. *J. Finance* 47 427–465. 10.1111/j.1540-6261.1992.tb04398.x

[B33] Foo Y. B. Zain M. M. (2010). Board independence, board diligence and liquidity in Malaysia: A research note. *J. Contemp. Account. Econ.* 6 92–100. 10.1016/j.jcae.2010.10.001

[B34] Garvey G. T. Swan P. L. (1994). The economics of corporate governance: Beyond the Marshallian firm. *J. Corp. Finance* 1 139–174. 10.1016/0929-1199(94)90001-9

[B35] Ghazizadeh P. Peek E. Rösch D. (2021). *Transparency and Liquidity in a Multi-Market Setting.* Available online at: https://ssrn.com/abstract=3852831 10.2139/ssrn.3852831 (accessed May 25, 2021).

[B36] Glosten L. R. Milgrom P. R. (1985). Bid, ask and transaction prices in a specialist market with heterogeneously informed traders. *J. Financ. Econ.* 14 71–100. 10.1016/0304-405X(85)90044-3

[B37] Grant R. M. (1996). Toward a knowledge-based theory of the firm. *Strat. Manag. J.* 17 109–122. 10.1002/smj.4250171110

[B38] Hodgson G. M. (2006). What are institutions? *J. Econ. Issues* 40 1–25. 10.1080/00213624.2006.11506879

[B39] Hooper V. Sim A. B. Uppal A. (2009). Governance and stock market performance. *Econ. Syst.* 33 93–116. 10.1016/j.ecosys.2009.03.001

[B40] Islam M. S. (2020). Foreign Currency Bond as Solution to Trade Deficit Induced Liquidity Crisis in Banks: Evidence from Bangladesh. *ILIRIA Int. Rev.* 10 89–106.

[B41] Jiraporn P. Chatjuthamard P. Tong S. Kim Y. S. (2015). Does corporate governance influence corporate risk-taking? Evidence from the Institutional Shareholders Services (ISS). *Finance Res. Lett.* 13 105–112. 10.1016/j.frl.2015.02.007

[B42] Jiraporn P. Kim J. C. Kim Y. S. (2011). Dividend payouts and corporate governance quality: An empirical investigation. *Financ. Rev.* 46 251–279. 10.1111/j.1540-6288.2011.00299.x

[B43] Karmani M. Ajina A. (2012). “Market stock liquidity and corporate governance,” in *29th International Conference of the French Finance Association (AFFI)*, (Virginia, US: Frozen Food Institute)

[B44] Katselas D. Sidhu B. K. Yu C. (2021). Liquidity and information asymmetry around unscheduled mining announcements. *Account. Finance* 61 3053–3087. 10.1111/acfi.12694

[B45] Kaufmann D. Kraay A. Mastruzzi M. (2009). *Governance matters VIII: Aggregate and individual governance indicators 1996-2008.* 10.1596/1813-9450-4978 Washington, DC: The World Bank.

[B46] Kennedy G. A. (2003). *Classical rhetoric and its Christian and secular tradition from ancient to modern times.* Chapel Hill, US: Univsity of North Carolina Press.

[B47] Krishnan S. Teo T. S. (2012). Moderating effects of governance on information infrastructure and e-government development. *J. Am. Soc. Inf. Sci. Technol.* 63 1929–1946. 10.1002/asi.22660

[B48] La Porta R. Lopez-de-Silanes F. Shleifer A. Vishny R. W. (1997). Legal determinants of external finance. *J. Finance* 52 1131–1150. 10.1111/j.1540-6261.1997.tb02727.x

[B49] Langbein L. Knack S. (2010). The worldwide governance indicators: Six, one, or none? *J. Dev. Stud.* 46 350–370. 10.1080/00220380902952399

[B50] Lei Q. Lin B. Wei M. (2013). Types of agency cost, corporate governance and liquidity. *J. Account. Public Policy* 32 147–172. 10.1016/j.jaccpubpol.2013.02.008

[B51] Lesmond D. A. Ogden J. P. Trzcinka C. A. (1999). A new estimate of transaction costs. *Rev. Financ. Stud.* 12 1113–1141. 10.1093/rfs/12.5.1113

[B52] Leuz C. Nanda D. Wysocki P. D. (2003). Earnings management and investor protection: An international comparison. *J. Financ. Econ.* 69 505–527. 10.1016/S0304-405X(03)00121-1

[B53] Levine D. K. (1998). Modeling altruism and spitefulness in experiments. *Rev. Econ. Dyn.* 1 593–622. 10.1371/journal.pone.0196524 29718977 PMC5931496

[B54] Lien Y.-C. Piesse J. Strange R. Filatotchev I. (2005). The role of corporate governance in FDI decisions: Evidence from Taiwan. *Int. Bus. Rev.* 14, 739–763.

[B55] Liu W. (2006). A liquidity-augmented capital asset pricing model. *J. Financ. Econ.* 82 631–671. 10.1016/j.jfineco.2005.10.001

[B56] Liu Y. Du H. Liu L. Leng J. (2014). Shape memory polymers and their composites in aerospace applications: A review. *Smart Mater. Struct.* 23:023001. 10.1088/0964-1726/23/2/023001

[B57] Liu Y. Miletkov M. K. Wei Z. Tina Y. (2015). Board independence and firm performance in China. *J. Corp. Finance* 30 223–244. 10.1016/j.jcorpfin.2014.12.004

[B58] Mbanyele W. Wang F. (2022). Board interlocks and stock liquidity: New evidence from an emerging market. *Emerg. Mark. Finance Trade* 58 1415–1429. 10.2139/ssrn.3775283

[B59] Méon P.-G. Weill L. (2005). Does better governance foster efficiency? An aggregate frontier analysis. *Econ. Gov.* 6 75–90. 10.1007/s10101-004-0080-z

[B60] Meyer J. W. Rowan B. (1977). Institutionalized organizations: Formal structure as myth and ceremony. *Am. J. Sociol.* 83 340–363. 10.1086/226550

[B61] Náplava R. (2018). “The Importance of Institutional Quality for Economic Performance in Post-Soviet States,” in *The International Scientific Conference INPROFORUM 2017*, (Ceske Budejovice: INPROFORUM), 159–164.

[B62] Nguyen C. T. Hai P. T. Nguyen H. K. (2021). Stock market returns and liquidity during the COVID-19 outbreak: Evidence from the financial services sector in Vietnam. *Asian J. Econ. Bank.* 5 324–342. 10.1108/AJEB-06-2021-0070

[B63] North D. C. (1991). Institutions. *J. Econ. Perspect.* 5 97–112. 10.1257/jep.5.1.97

[B64] Pett M. A. Lackey N. R. Sullivan J. J. (2003). *Making sense of factor analysis: The use of factor analysis for instrument development in health care research.* 10.4135/9781412984898 California, US: Sage.

[B65] Porta R. L. Lopez-de-Silanes F. Shleifer A. Vishny R. W. (1998). Law and finance. *J. Political Econ.* 106 1113–1155. 10.1086/250042

[B66] Prommin P. Jumreornvong S. Jiraporn P. (2014). The effect of corporate governance on stock liquidity: The case of Thailand. *Int. Rev. Econ. Finance* 32 132–142. 10.1016/j.iref.2014.01.011

[B67] Raheja C. G. (2005). Determinants of board size and composition: A theory of corporate boards. *J. Financ. Quant. Anal.* 40 283–306. 10.1017/S0022109000002313 8344824

[B68] Rajan R. G. Zingales L. (1998). Power in a Theory of the Firm. *Q. J. Econ.* 113 387–432. 10.1162/003355398555630

[B69] Schwartz R. A. Francioni R. Weber P. (2020). Market Liquidity: An Elusive Variable. *J. Portf. Manag.* 46 7–26. 10.3905/jpm.2020.1.174

[B70] Shleifer A. Vishny R. W. (1997). A survey of corporate governance. *J. Finance* 52 737–783. 10.1111/j.1540-6261.1997.tb04820.x

[B71] Smith A. (1776). *The wealth ofnations.* New York, NY: The Modern Library.

[B72] Tarchouna A. Jarraya B. Bouri A. (2017). How to explain non-performing loans by many corporate governance variables simultaneously? A corporate governance index is built to US commercial banks. *Res. Int. Bus. Finance* 42 645–657. 10.1016/j.ribaf.2017.07.008

[B73] Teece D. J. (1986). Profiting from technological innovation: Implications for integration, collaboration, licensing and public policy. *Res. Policy* 15 285–305. 10.1016/0048-7333(86)90027-2

[B74] Tricker R. B. Tricker R. I. (2015). *Corporate governance: Principles, policies, and practices.* Oxford: Oxford University Press.

[B75] Utami W. Wahyuni P. D. Nugroho L. (2020). Determinants of Stock Liquidity: Forward-Looking Information, Corporate Governance, and Asymmetric Information. *J. Asian Finance Econ. Bus.* 7 795–807.

[B76] Wahed M. S. (2017). How successfully China has applied the OECD principles of corporate governance: A critical assessment. *J. Chin. Econ. Bus. Stud.* 15 353–372. 10.1080/14765284.2017.1371562

[B77] Wang F. Mbanyele W. Muchenje L. (2022). Economic policy uncertainty and stock liquidity: The mitigating effect of information disclosure. *Res. Int. Bus. Finance* 59:101553. 10.1016/j.ribaf.2021.101553

[B78] Weill P. (1992). The relationship between investment in information technology and firm performance: A study of the valve manufacturing sector. *Inf. Syst. Res.* 3 307–333. 10.1287/isre.3.4.307 19642375

[B79] Yang T. Zhao S. (2014). CEO duality and firm performance: Evidence from an exogenous shock to the competitive environment. *J. Bank. Finance* 49 534–552. 10.1016/j.jbankfin.2014.04.008

